# Research collaboration with care home residents: a systematic review of public involvement approaches

**DOI:** 10.1186/s40900-025-00724-0

**Published:** 2025-05-15

**Authors:** Megan Davies, Lisa Irvine, Tamara Backhouse, Poppy Carr, Elspeth Mathie, Michelle Drury-Mulholland, Gizdem Akdur, Anne Killett

**Affiliations:** 1 School of Health Sciences, Faculty of Medicine and Health Sciences, University of East Anglia, Norwich, NR4 7TJ UK; 2 Centre for Research in Public Health and Community Care (CRIPACC), University of Hertfordshire, College Lane, Hatfield, AL10 9AB UK; 3 Skills for Care, 6 Grace Street, Leeds, LS1 2RP UK

**Keywords:** Public Involvement, PPI, Older Adults, Long-Term Care, Care Homes

## Abstract

**Background:**

Public involvement is crucial to ensure research is relevant and addresses the needs of its target population. However, care home residents, a potentially vulnerable group, are often excluded from research that could directly benefit them. This systematic review examined the existing literature on public involvement approaches in research involving older adults residing in long-term care homes.

**Methods:**

A systematic search of CINAHL, MEDLINE and PsychINFO was conducted, using search terms related to public involvement and long-term care. The search was limited to English language papers published from 2014, building on a 2016 review conducted by Backhouse et al. Articles were screened by title and abstract, and full texts of potentially eligible papers were reviewed for inclusion. Data from included studies was extracted and synthesised using a narrative approach.

**Results:**

This review identified 15,809 citations, abstract-screened 4000, and ultimately included six articles after applying eligibility criteria and a rigorous screening process. Reported public involvement in this setting was limited, with even fewer studies demonstrating genuine collaboration and the full involvement of residents throughout the research process. There was a lack of representation of residents with advanced cognitive decline or dementia. Terminology used to describe public involvement varied considerably across studies, highlighting a lack of clarity in defining and reporting activities.

**Conclusions:**

This review highlights the need for greater emphasis on public involvement in care home research, particularly for residents with cognitive impairments. Future research should prioritise transparent reporting of public involvement processes, involving residents as active partners from the outset, and ensuring research findings are effectively communicated for all stakeholders, including residents. Barriers and facilitators to public involvement activities in care homes are summarised.

**Supplementary Information:**

The online version contains supplementary material available at 10.1186/s40900-025-00724-0.

## Background

Successful research in long-term care, such as nursing or care homes, can enable value-based decisions and inform improvements to policy and care practices [1]. This can include the understanding of information gained from speaking to residents, staff, family members and the wider community. Recent research has shown the importance of including a range of long-term care residents, including those living with cognitive decline or dementia, which account for up to 80% of residents in long-term care [2], in research practices [3]. Long-term care is a ‘unique and complex environment’ and it is important that the experiences of people living within the environment are considered in research [4]. Despite this, long-term care residents, particularly those living with a level of cognitive decline remain an under-represented population in long-term care research [5, 6]. Using more adaptable methods can facilitate research with residents in long-term care [7]. This includes the overall methodology, for example using ethnographic techniques, which allows for informal conversations during naturally occurring events and immersion within an environment during periods of data collection [8]. This also includes restructuring research relationships, to include residents and other key stakeholders’ input throughout the whole research process, using methods such as co-production, participatory action research (PAR), patient and public involvement (PPI) and patient and public involvement and engagement (PPIE). Involving a full range of stakeholders, and residents in long-term care research is important to enhance the quality and the relevance of the research [3, 9, 10].

While the term PPI is widely used, this paper focuses on the involvement of older adults residing in long-term care homes. Given the setting and the holistic nature of care within these environments, where residents may not always primarily identify as'patients'in a medical context, we have chosen to use the term'public involvement'to encompass the broad range of residents'perspectives and experiences. This aligns with the understanding that long-term care is a ‘unique and complex environment’ where the experiences of all people living within it are important to consider in research. A spectrum of public involvement exists in research, with some highlighting co-production as an ‘ideal’ practice to be included [11]. Co-producing research means involvement is incorporated throughout the entirety of the project, including consultation, protocol development and review, input to manuscripts, feedback on research questions, participant materials, editing survey questions, consent forms, information sheets and summaries for readability and providing assistance with recruitment, public engagement and dissemination [11]. Co-production, along with some other research practices on the public involvement spectrum, involves close collaboration, sharing of power and knowledge and levels of respect and reciprocity between researchers, practitioners and the public, which can lead to lasting relationships [12]. Also noted as part of the public involvement research spectrum is PAR, which focuses on bringing together a group of people to help improve or solve a known problem with shared relevance. Here, a partnership is created to generate, test, and provide feedback on the solution [13].

Several existing reviews highlight effective implementation of public involvement. Backhouse et al. [3] reviewed the feasibility of public involvement with older adults living in long-term care homes and concluded that older residents can be successfully involved in the research process, but there were few examples of care home research where this was achieved. Typically, the studies that achieved more collaborative public involvement practices were smaller scale studies with public involvement practices in larger scale studies confined to advisory roles. Baldwin et al. [14] reviewed research involving older adults in public involvement in health and social care research. Their study concluded a need for future consideration of older adult’s skills and motivation when matching individuals to a project as well as the individual’s level of involvement and embedding evaluation into an iterative research process. Both reviews successfully incorporated their own public involvement through reviewing the themes with public involvement team members [3] and advisory group participants [14].

Price et al. [15] acknowledged limitations stemming from the lack of standardised public involvement reporting guidance and structure, which could have caused missed public involvement reporting in literature. In general, continued under-reporting of public involvement, has been found by Gray et al. [16] as an ongoing issue. None of the included randomised control trials within their research reported on public involvement activities, meaning they were unable to identify differences recorded in public involvement reporting [16]. More recent research by Lang et al. [17] suggested future research could focus on the “quality of [public involvement] reporting, perhaps based on established criteria” [17]. In 2023, a review by Burgher et al. found that implementing a person-centred approach to public involvement could improve stakeholder involvement at different levels. Communication barriers across staff and resident groups in public involvement sessions, for example during the set-up of activities, can make public involvement challenging. In addition, they noted that a gap in including residents with higher needs in terms of functional or cognitive decline was still evident. The additional needs and often time pressured studies presented challenges in bridging this gap in research, which still needs to be addressed [9]. It should be noted that while resident voices have been included within previous reviews, they were involved to varying degrees within the included papers, with many articles still including no resident voices. The evidence-based public involvement guidelines for future long-term care home research were therefore not specifically tailored to encourage resident involvement [9]. Overall, it is evident that research papers often report on the process of public involvement more heavily than the impact of the public involvement, which not only hinders learning about the benefits, but risks acceptance of tokenistic public involvement practices [18]. Furthermore, despite a noticeable increase in the number of public involvement papers being published in recent years, challenges in public involvement still persist [19].

Previous research, including Backhouse et al. [3] and Burgher et al. [9], has contributed to the understanding of public involvement in long-term care. This systematic review provides a necessary update to account for more recent literature and has a specific focus on the collaborative involvement and experiences of care home residents themselves. This review aimed to understand approaches, successes, barriers and facilitators of public involvement activities for older adults residing in long-term care homes, in research published between 2014 and 2023, including:

- The latest key research messages from successful collaborative research involving older adults living in long-term care homes,
- Successes in public involvement with older adults in care home settings,
- The skills, resources, and planning needed and who the key stakeholders are
- Potential barriers and facilitators to public involvement activities that include older adults living in long-term care homes.

## Main text

### Methods

A systematic review was conducted following guidance from the PRISMA 2020 checklist [20]. This includes developing informed research questions, extracting selective and relevant data from documents and synthesising data in the final review using appropriate techniques.

#### Eligibility criteria

Studies were included if they were primary studies. All studies had to include older adults (> 65) residing in long-term care homes (including care homes, nursing homes or any residential facility providing 24-h services or care to older adults). The older adults had to have been included in public involvement or a public involvement variant activity. Only English language papers were included.

#### Information sources and search strategy

Online databases CINAHL (EBSCO), MEDLINE (Pubmed) and PsychINFO (OVID) were systematically searched using two facets including variants of 1) Public Involvement in Research and 2) Long-Term Care. Boolean operators AND and OR were used to combine search terms. For full list of Information Sources see Appendix 1. Phrase searches, proximity operators and truncation were also used. All terms were searched for by title and abstract. Limits were set to 2014, which was the year in which searches from the paper being updated [3] were completed. Controlled vocabulary terms were used when provided by the database. In addition, searches of NHS/NICE evidence, the Cochrane Library and Google Scholar were conducted. Snowballing (searches of references within papers that have cited Backhouse et al., [3] and Burgher et al., [9] was used to identify further potential papers for inclusion. Papers found through snowballing were cross-referenced with papers included from the initial database search to remove duplicates.

#### Selection process

All citations were exported to EndNote X9. One reviewer (MD) removed all duplicates in the first instance. Papers for inclusion were then dual screened by title and abstract and following this screened by full text. This was undertaken independently by two reviewers (MD and LI), and any discrepancies were resolved through discussion.

#### Data collection process

Data was extracted by two reviewers. Data to be extracted was discussed and decided with input from the lead author on the review paper being built upon (TB) to ensure this paper would bridge any gaps in knowledge. Any uncertainties and discrepancies were discussed. Extracted data from included papers was input into a Microsoft Excel spreadsheet to record details. As this is a systematic review, we used PRISMA to guide our reporting. The lack of GRIPP2 (international guidance for reporting of patient and public involvement in health and social care research) in the primary studies means there is potentially less transparent justification of the public involvement processes within those individual studies. While GRIPP2 could have provided a more standardised framework for *what* to report in primary studies about public involvement, this review had to work with the information that was actually reported. The variability in terminology and the lack of detailed reporting in the included studies made a comprehensive extraction based on GRIPP2 principles challenging, as that information was often missing.

#### Data items

Data items for extraction were author name, publication year, methodology, title, country, public involvement definition and resident involvement in public involvement, stages of research, how the process was closed/ended, barriers, facilitators, remuneration and reasons for resident exclusion from studies.

#### Synthesis methods

All study characteristics, including stages of research and methods for public involvement were tabulated using Microsoft Excel. From this, the two reviewers were able to determine the eligibility for inclusion for each study. Full study characteristics of included papers can be found in Table 1. A narrative synthesis output was selected as a means of further explaining and presenting critical but trustworthy information (Popay et al., 2006).

**Table 1:** Summary of Included Studies

**Author and Publication Year**	**Country**	**Aim of study**	D**efinition of Public Involvement Activities**	**Methodology**	**People involved**	**How were residents engaged? (e.g. pictures, 1:1, focus group etc.)**
de Boer et al. [21]	Netherlands	Co-creation of an alternative nursing home model ("the Homestead")	Participatory Research Approach to co-creation	Case study design using PAR methodology	Older adults, family members/representatives, care staff, management, architects, and design staff	Not specified
Giné-Garriga et al. [22]	Scotland and Spain	Co-create interventions to reduce sedentary behaviour in care home residents	Co-creation and PAR	Qualitative intervention using PAR methodology	Residents, University students, researchers, staff members, family members and policy makers	Workshops/focus groups—no further description provided
Luijkx et al. [23]	Netherlands	Describes new collaboration between science, care practice, and education designed to improve long-term care for older adults	‘Joining Forces', evidence-based knowledge, co-creation, collaboration	Implementation process and intervention	Academics (various levels/backgrounds), communication, education and implementation experts, care professionals, older adults, teachers	Interviews and interactive meetings using'creative work forms', e.g. board games. Approaches to communication are managed by the designated'communication expert'
Hemphill et al. [24]	Canada	Describes a Quality Improvement initiative within a long-term care organisation	Stakeholder engagement, co-creation, and stakeholder groups	Mixed method consultation—surveys and small group conversations	Older adults, family members, care home staff, local authority staff	fact-gathering conversations, stakeholder survey, half-day stakeholders meeting
Petriwskyj et al. [25]	Australia	Explore understandings and practices of engagement within an aged-care organisation from both staff and client viewpoints, examining the extent of client power	Engagement	theoretical paper	Care staff, Older adults ('clients') across residential, community and retirement living settings	semi-structed interviews, focus groups
Woelders and Abma [26]	Netherlands	Presented ways enhance the collective involvement of care home residents, including power dynamics	Participatory Research Approach	Qualitative	Older adults and facilitators (spiritual counsellors)	semi-structured interviews and reflection

#### Study risk of bias assessment

The aim of the systematic review was to explore the landscape of public involvement approaches in long-term care research, identify successful practices, barriers, and facilitators, and update a previous review. This is inherently a process-focused endeavour. We are interested in understanding how public involvement was being conducted and reported, not in evaluating the effectiveness of research interventions in care homes. Therefore, applying tools designed to assess bias in effect estimates would not be an appropriate methodological choice for the review question [27].

## Results

### Study selection

**Fig. 1:**
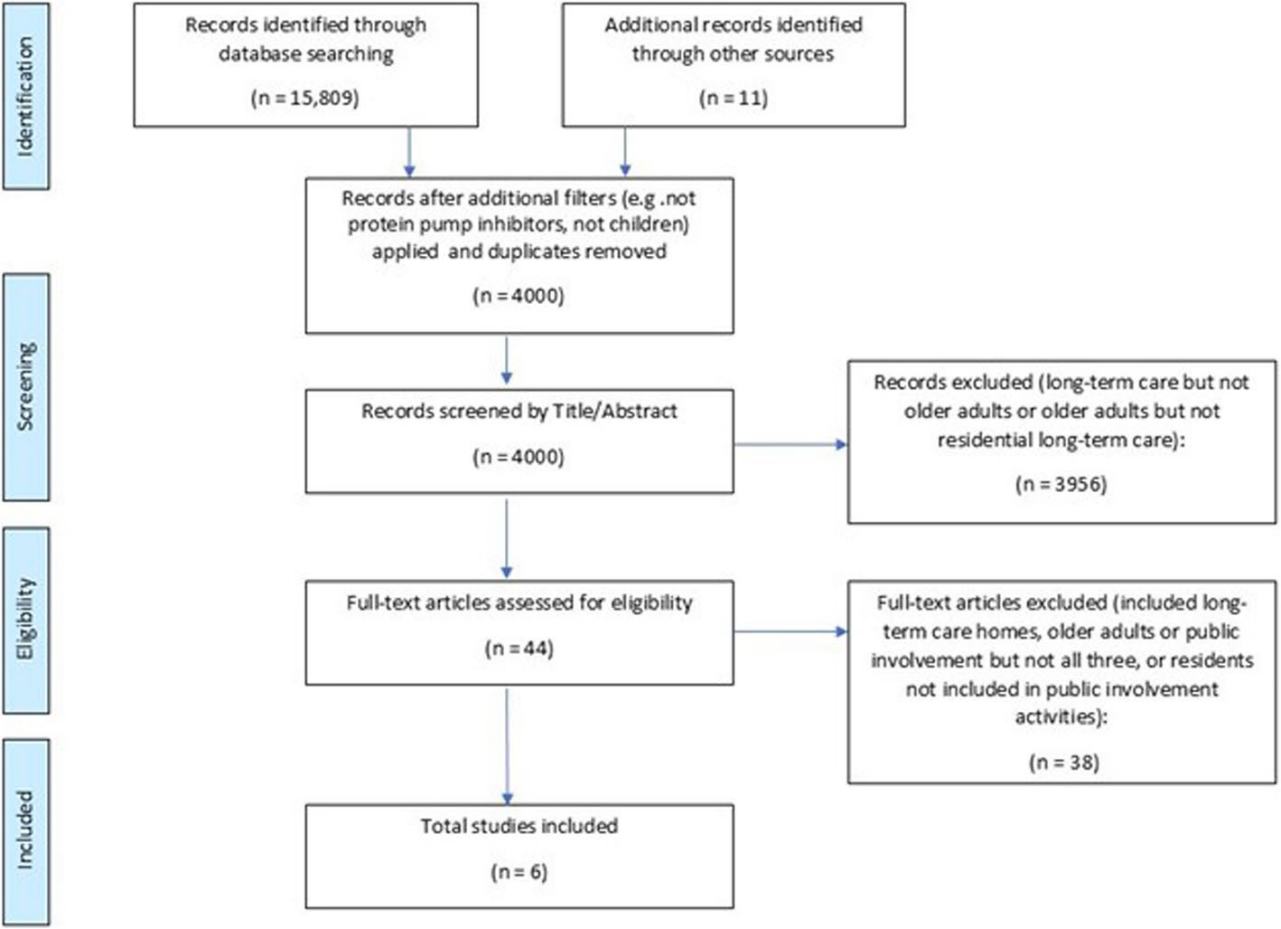
PRISMA flow diagram

**Fig. 2:**
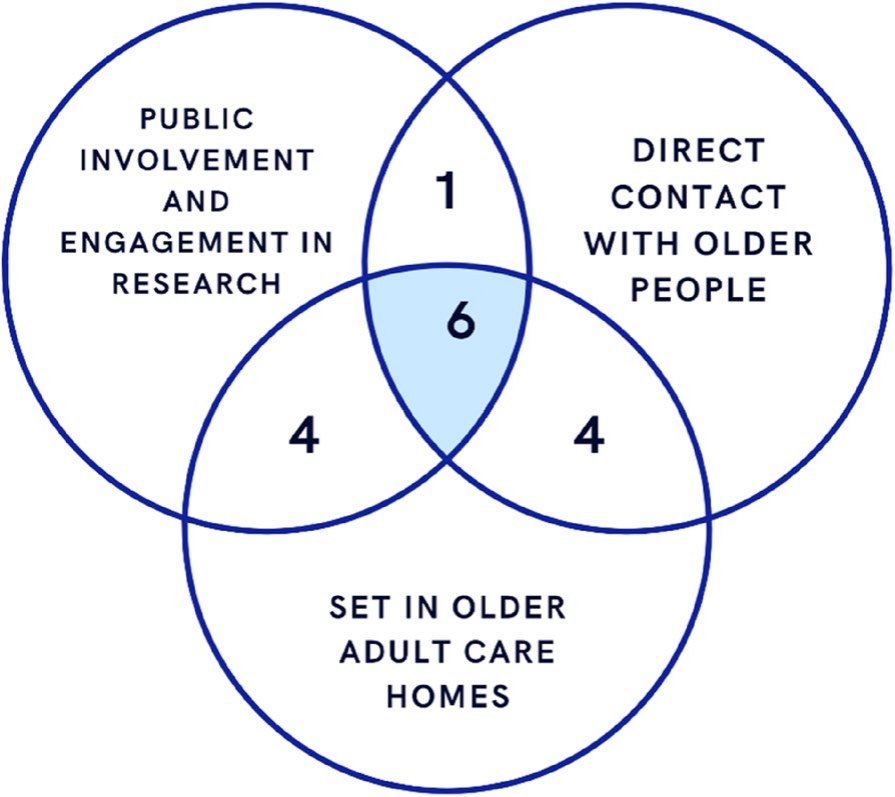
Venn diagram of included studies based on Table 4

The search initially identified 15,809 citations (see Fig. 1 PRISMA Flow Diagram). Following the initial search, it was clear that there was a lack of sensitivity in the original search strategy. For example, ‘PPI’ as a term picked up ‘Protein Pump Inhibitors’ and conference proceedings and children’s/learning disability care homes were also retrieved in the search. Therefore, further filters were applied to ensure only relevant papers remained, which left 4000 papers to be screened by title and abstract after duplicates were also removed. After title and abstract screening took place, 44 papers were screened by full text for inclusion, of which 15 were initially selected for inclusion. All 15 had at least 2 of the following: older adults, public involvement, or took place in a care home however, after reviewer discussion, only six articles were found to meet the full inclusion criteria (see Fig. 2).

Study designs comprised a combination of qualitative, quantitative and mixed methods research. For the most part, included papers benefitted from some level of qualitative information, which included data collection methods as well as critical reflections. Most of the studies were conducted in the Netherlands (three studies), with one taking place across Scotland and Spain, one in Canada and one in Australia. All studies stated a focus on including care home residents in public involvement activities.

## Synthesis of results

### Public involvement activities as defined by authors

The included papers span the spectrum of public involvement techniques, using different terminology and descriptions to achieve a similar goal (see Table 2). Multiple terms were described throughout the papers, including Participatory Research, Co-creation, Stakeholder Engagement and Collaboration. The broad range of terminology included demonstrates the complexity and potential for vague descriptions within public involvement techniques.

**Table 2:** Paper definitions of Public Involvement activities

Author(s)	Public Involvement Terminology Used	Description of Terminology
De Boer et al., [21]	Participatory Research Approach to co-creation	Key stakeholders (here this is older people, their families and representatives, long-term care staff, management, architects and design staff) working with researchers to put scientific knowledge on how to design the physical, social and organizational environment in everyday care into practice
Giné-Garriga et al., [22]	Co-creation and PAR	Working with stakeholders to design an intervention for behaviour change
Luijkx et al., [23]	‘Joining Forces', evidence-based knowledge, co-creation, collaboration	Collaboration with multiple stakeholders
Hemphill et al., [24]	Stakeholder engagement, co-creation, and stakeholder groups	Involving stakeholders by gathering feedback and perspectives.
Petriwskyj et al., [25]	Engagement	Engagement incorporates a broad range of activities, including those designed to inform or educate, gather information or consult, discuss or involve, collaborate on a more equal footing, or empower clients
Woelders & Abma [26]	Participatory Research Approach	Collaboration inspired by response evaluation – attempts to involve all people in a certain setting

### People involved in public involvement activities

All papers presented results addressing resident voices within the respective studies. However, only three papers within this review put the resident voices at the forefront of the results [22, 25, 26]. Furthermore, these papers still included a combination of older adults’ voices with other stakeholders in the results. Within the papers engaging multiple stakeholders within long-term care home communities, the number of residents included were notably lower than other groups. In general, all included studies seemed to be inclusive of all long-term care residents, although they remained low in participant number across the papers. It was common in all papers for researchers to state no reason for not including residents in public involvement activities. Although, Giné-Garriga et al. [22] specifically noted that while no residents were excluded due to health reasons, residents were recruited based on their assumed ability to hold discussions with the student researchers involved in the study.

### Recruitment processes

Not all studies were clear on the recruitment process. In some cases, the care organisation or individual care home had partnered with a university or was already part of an ongoing collaborative project, where the whole care home had been recruited [22, 23]. This meant that the justification for and actions towards the recruitment process only outlined that of the care home rather than recruitment of individual residents.

### Stages of resident involvement and means of engagement

Resident involvement within each of the research stages varied across the included papers. For the most part, residents were reported to have been involved in the early stages of the research process, which was reflected in successful research designs [21–23]. This included development of care models and designing the research topic. Involvement for these papers continued throughout the research, with residents participating in interactive activities such as workshops, interviews, surveys and focus groups. One study [26], which specifically referred to using PAR did not include older adults in the design or set up of their study, instead including them only in the PAR groups. This was also seen from de Boer et al. [21], who did not include older adults in the ‘core development’ stage, instead including older adults in working groups for feedback. All studies failed to report dissemination of information to older adults participating or whether any debrief session took place at the end of the studies. It was reported that this tended to leave older adults feeling dissatisfied rather than having a positive experience of information shared.

Older adults were engaged in public involvement activities in a variety of ways. Some studies mentioned workshops/focus groups/stakeholder meetings or semi-structured interviews with no elaboration on what this looked like. Others described ‘creative work forms’ used, such as board games, interactive meetings and role play. The more creative tools used were found to be successful in facilitating engagement and communication with a study facilitator or ‘communication expert in place’, but staff shortages and knowledge gaps in day-to-day use hindered their use beyond the specific research being conducted.

### Remuneration

Remuneration was not discussed in any of the included papers. Although, it is not possible to confirm that researchers did not follow up with residents after the specific papers had been published as some were described as an ‘ongoing collaboration’ [21, 23].

### Barriers and facilitators to public involvement activities

Table 3 shows the main barriers and facilitators to Public Involvement activities with care home residents from this review and the previous Backhouse et al. review. Recent data showed realistic outcomes of care organisations over-ruled expected or hoped for outcomes. Having an ‘implementation expert’ as part of one study to assess the readiness to use intended tools within an individual organisation helped to overcome this [23]. Regardless of outcomes, buy-in from care home staff and management was noted as either a barrier or facilitator depending on the levels of enthusiasm, with higher buy-in acting as facilitators and lower becoming barriers to Public Involvement activities. Including internal facilitators, varied backgrounds among the research team and stakeholder groups and the research group being known to the care home/organisation helped with both communication and buy-in. External facilitators were found in more than one paper to increase rigour and candidness among older adults joining public involvement activities. Although, researchers with less experience in involving older adults in research were noted as a barrier. Resident health issues (specifically residents living with mild to moderate dementia) and resident tiredness/loss of concentration during focus groups were noted in one paper as a barrier to obtaining information. Additionally, miscommunication and a lack of confidence in residents were also noted as barriers. In general, a lack of evaluation of implementation processes/public involvement activities made it difficult to clearly outline the barriers or facilitators.

**Table 3:** Factors that could be barriers to or facilitators of residents’ involvement in research processes – adapted from Backhouse et al. [3]. New items from this review in bold and with ^*^, items evident in both reviews in bold and with ^**^

		**Barrier and facilitator categories**				
	**Social factors**		**Skills**	**Resources**	**Care-home organisational factors**	**Organisation of the research**
**Barriers**	-Resident low confidence^**^ -Apprehension to engage into something different -Power relations (in relation to staff and relatives) -Researcher and research seen as threatening (to staff) -Frustration about complexity and slow progress -Lack of trust in confidentiality -Low or changing mood of some residents -Role conflict of researching in own home		-Sensory and communication difficulties -Changing resident health^**^ -Cognitive impairment^**^ resulting in limited participation/negotiation skills -Meetings monopolised by one member -Lengthy and complex reports frustrating residents -Residents’ low energy/tiredness^*^ -Loss of resident concentration^*^ -Lack of experience leading co-creation (students)^*^ -Gaps in knowledge^*^	-Lack of funding for more continuous input -Limited researcher time (e.g. not available at the weekend, no time for providing feedback) -Lack of space to hold meetings	-Unsupportive organisational culture -Care organisation over-ruling expected outcomes^*^ -Individuals and groups feeling isolated from each other -Perception that residents'involvement might slow down decision-making process -Dominant person might influence residents -COVID 19 exacerbated stigmas and need for culture change^*^	-Limited researcher flexibility -Ethical protocols excluded and limited participation -Researchers reluctance to relinquish control -Timing of meetings e.g., evening -Venue of meeting e.g., not at care home or lack of privacy -Miscommunication^*^ -No evaluation of implementation, so no identification of barriers^*^
**Facilitators**	-Development of trust and good relationships -Residents’ experiences valued -Residents supported to contribute -People open to change -Good commitment from public involvement members -Transparency of processes -Residents having some control e.g. ownership of decisions -Assurance that the study will result in progress -Assured confidentiality -Assured withdrawal at any time without reason		-Constant encouragement and support of residents from researchers -Researchers embracing deviant perspectives -Researchers using successful examples to illustrate involvement -Researchers willing to share control -Researchers always contactable -Negotiated ground rules -Ability to communicate with diverse groups of people^**^ -Use of creative methods to engage residents -Researchers being flexible -Separate workshops for staff or family members^*^	-Funding for honorarium for participants -Time to do the groundwork required, e.g. proving information -Time to arrange meetings and support residents -Suitable venues and space to hold meetings -Providing sustenance -Financial resources to implement changes identified by the research -Independent university researchers facilitated Public Involvement^*^ -External team lead the initiative—capacity for candidness^*^	-Supportive organisational culture -Care-home management on board -Care-home management willing to change -Care-home staff value residents being involved in study -Research group known to care organisation^*^ -Expert assesses whether individual organisation is'ready'to use the tool intended for implementation^*^	-Emergent study design -Use topics that really matter to the residents -Flexibility in residents’ involvement, e.g., informal conversations -Allow personal ad hoc contact with research team -Summarise meeting notes into accessible formats, e.g. posters -Send materials out before meetings -Recruit researchers who can support older people -Recognise multiple stakeholder groups/support marginalised groups -Diverse range of stakeholders/backgrounds^*^ -Outline key implementation changes in an accessible way^*^

## Discussion

### Summary of findings

This systematic review aimed to examine the public involvement reported in studies taking place in care homes and involving older adults. It aimed to explore the latest key research practice messages from collaborative research involving older adults living in long-term care homes, the barriers and facilitators to this and the skills, resources and planning needed. Multiple terms to describe public involvement have been used across the included studies, with limited expansion on what public involvement activities actually looked like in practice. Resident voices in public involvement were acknowledged as the focus, although half of the included papers still failed to put them at the forefront or standalone of results presented. Furthermore, even where residents were reportedly involved in all stages of the research/public involvement process, dissemination practice and remuneration were omitted. The included information was synthesised to explore these aspects.

### Persistent gaps in reporting and collaborative involvement

Limited research reporting public involvement activities in research involving care homes was found, and those involving older adults were even fewer. This was also found in the review preceding this by Backhouse et al. [3], with literature searches concluded 9 years prior to those completed in this review. Coproduction and public involvement with care home residents in research activities in the health and social care sector goes beyond consulting residents and power should shift towards the end users [28, 29]. Despite coproduction and public involvement activities being described in the included papers, involvement of residents from start to end of the research being conducted was seen in few. Furthermore, there was a distinct lack of residents who were living with advanced cognitive decline or dementia mentioned in the included papers, which reinforces previous research highlighting the underrepresentation of such residents in research activities [5, 6]. In theory, it is possible for all health and social care research to include public involvement activities with residents, including residents living with dementia. Practically, however, accessibility, health and safety and ethical concerns surrounding potential residents to be included in public involvement activities still limits the level of inclusivity seen. Woelders and Abma [26] introduced named facilitators, who were found to improve resident involvement in research activities, particularly facilitators who were known members of the care home community, such as spiritual counsellors. This gave residents a platform that encouraged information sharing and problem solving. However, gaps between facilitators and care teams were still evident, which prevented information from residents reaching care teams. Participatory processes increasingly enable resident voices in care home research and the facilitators known to residents stand to enable coproduction and the inclusion of resident voices within the context of a care home setting [30]. Failure to share information that comes from care home residents, as well as a failure to share research results, can leave residents feeling frustrated and underappreciated. Despite this being acknowledged within the included papers, no clear follow up was mentioned.

There is a shortage of public involvement reported in wider health and social care research, but it appears there has been an acceptance of the need to report limitations and the shortcomings of public involvement activities [31]. Furthermore, reported public involvement activities have low participation rates, or none have been included in the final results presented [9].

It has been found that papers largely ignore issues of power dynamics and disengagement from the public involvement or provide more tokenistic attempts to involve patients or the public. Price et al. [15] concluded that the shortage of public involvement reporting may also be due to the aversion to report unsuccessful public involvement or absence of public involvement work to report on.

### Limitations of this review

This review was limited by the inclusion of only English language papers due to language restrictions within the team, which may have restricted access to research presented across a broader range of countries. With this limit set, most research presented came from the Netherlands and then from English speaking countries. There was a lack of reporting on public involvement activities, and in fact, some public involvement methodology described were actually research methods, potentially blurring roles between public involvement activities and participants (see Table 4). Therefore, we may not know the full involvement of care home residents within the studies presented and also may have missed other research including resident Public Involvement due to lack of reporting. The quality of the included studies'methodologies might influence the robustness of the reported information on public involvement, even though it wasn't the primary focus of their appraisal.

**Table 4:** Papers meeting inclusion criteria

**Reference (Vancouver)**	**Paper**	*Direct contact with older adults (care home residents residents) reported (OA)*	*Public involvement and engagement in research (not just qualitative research) (PPIE)*	*Set explicitly in older adult care homes (CH)*	*How many inclusion criteria fulfilled*	*all*
x	**de Boer 2021**	Yes	Yes	Yes	3	**ALL**
x	**Giné-Garriga 2019**	Yes	Yes	Yes	3	**ALL**
x	**Hemphill**	Yes	Yes	Yes	3	**ALL**
x	**Luijkx**	Yes	Yes	Yes	3	**ALL**
x	**Petriwskyj**	Yes	Yes	Yes	3	**ALL**
x	**Woelders**	Yes	Yes	Yes	3	**ALL**
x	**Wherton**	Yes	Yes	**No**	2	PPIE & OA
x	**Bail**	Yes	**No**	Yes	2	OA & CH
x	**Casey**	Yes	**No**	Yes	2	OA & CH
x	**Harrison**	Yes	**No**	Yes	2	OA & CH
x	**Sion**	Yes	**No**	Yes	2	OA &CH
x	**Scheffelaar**	**No**	Yes	Yes	2	PPIE & CH
x	**Stocker**	**No**	Yes	Yes	2	PPIE & CH
x	**Walsh**	**No**	Yes	Yes	2	PPIE & CH
x	**Willis 2018**	**No**	Yes	Yes	2	PPIE & CH

### Comparison with findings from Backhouse – what has changed?

This review has added knowledge to the previous Backhouse et al. [3] review which it has updated. New evidence shows how residents have been involved, highlighting that resident involvement can be successful when facilitated by independent or external personnel, when they have specific involvement separate from other stakeholders, and when research groups have previous connections with care organisations. However, since the previous search conducted in 2014 only six articles explicitly report involvement of care home residents as public involvement collaborators or advisors. As was highlighted in the Backhouse et al. review, quantitative research involving residents is still lacking, as are larger studies. While public involvement reporting guidelines (GRIPP2) [32] have been developed, there are still reporting limitations in the included studies, which, in fact, did not report using GRIPP2. Evidence from both reviews demonstrates that older care-home residents can be successfully involved in research processes. This knowledge coupled with increased emphasis from research funders to involve people with lived experience in research, demonstrates that there is clear scope to augment the role of resident involvement. Researchers may feel wary of involving residents due to access and capacity challenges and care-home managers as gatekeepers may feel protective about resident involvement however, findings from both reviews (Table 3) outline clear facilitators for involving care home residents, which will be of practical use to researchers.

## Conclusions

Public involvement activities in care homes, including the terminology used for public involvement, remains broad and vague across studies, making it difficult to define and implement. A broader understanding of what public involvement within research means is needed to aid the identification of relevant papers. Regardless of how successful public involvement activities within research are reported to be, the description and amount of resident involvement is still lacking. Papers highlighting the use of public involvement activities as a strategy for research are still at risk of the tokenistic inclusion of residents, particularly those living with cognitive decline, regardless of steps taken to creatively facilitate inclusivity. Residents and care staff should be included as early as possible in research as part of a co-production design to facilitate the use of public involvement activities, improve resident confidence and increase staff buy-in. Further information on how residents are engaged and how activities are adapted to make them more inclusive for all residents, regardless of needs, is essential to successfully implement future public involvement activities. Future research should also focus on dissemination of results to residents and remuneration, which are currently under-reported.

## Supplementary Information

Supplementary Material 1.

## Data Availability

No datasets were generated or analysed during the current study.

## Abbreviations

PPI: Public and Patient Involvement
NIHR: National Institute for Health and Care Research
PAR: Participatory Action Research
NHS: National Health Service
NICE: National Institute for Health and Care Excellence
GRIPP2: International guidance for reporting of patient and public involvement in health and social care research
