# Supplementary material for: Research collaboration with care home residents: a systematic review of public involvement approaches
Source: Res Involv Engagem. 2025 May 15;11:49. doi: 10.1186/s40900-025-00724-0 (PMC12083167; doi:10.1186/s40900-025-00724-0)
Supplement: Supplementary file 1 — Supplementary Material 1. [file 40900_2025_724_MOESM1_ESM.docx]

*This systematic review examined how older adults residing in long-term care homes have been involved in research. Care home residents (especially those living with dementia) are often left out of research that affects their lives. Searching studies published since 2014, this review identified only six papers that explicitly reported using public involvement approaches like co-production or participatory research, out of over 15,000 initial citations found through searching databases. The six studies used different terms and definitions for public involvement, making it difficult to compare findings. This lack of clarity makes it harder to learn from what others have done. Reported public involvement activities were limited, with few studies demonstrating genuine collaboration or including residents throughout the entire research process. As known from previous research, a significant gap was the lack of inclusion of residents with advanced cognitive decline or dementia. While some studies used creative methods like interactive meetings to engage residents, involvement in stages like disseminating findings was rarely reported. Remuneration for residents was not mentioned.  Reported barriers to public involvement with care home residents included cognitive impairment, communication issues, changes to residents’ health, and a lack of consistent reporting or evaluation. The use of experienced or known facilitators who can build trust, supportive care home staff/management, and adapting methods to residents' needs assisted public involvement with residents. Overall, there is a strong need for more transparent reporting and more inclusive, and truly collaborative involvement of care home residents in research, particularly those with cognitive impairments, recommending early inclusion.*
